# Supplementary material for: Extreme primary and secondary protein structure variability in the chimeric male-transmitted cytochrome c oxidase subunit II protein in freshwater mussels: Evidence for an elevated amino acid substitution rate in the face of domain-specific purifying selection
Source: BMC Evol Biol. 2008 May 31;8:165. doi: 10.1186/1471-2148-8-165 (PMC2430956; doi:10.1186/1471-2148-8-165)
Supplement: Additional file 1 — Amino acid sequences deduced from the complete Mcox2 extension nucleotide sequences of the 21 species used in this study. [file 1471-2148-8-165-S1.pdf]

Additional Table 1. Amino acid sequences deduced from the complete M*cox2* extension nucleotide sequences of the 21 species used in this study (using the *Drosophila* mtDNA genetic code). Sites shaded in black are the 11 constant amino acid positions; the eight sites shaded with blue are those that may be under positive selection as inferred by the MG94xHKY85x3_4x2_Rates model in *HyPhy*; the six sites shaded with orange are those that may be under positive selection as inferred by the M8 model in the *codeml* algorithm in PAML. In the C-terminus tail regions, the N-glycosylation motifs are shaded in red, the casein kinase II phosphorylation motifs are shaded in green, and areas where these motifs overlap are shaded in blue.

```
                                 10        20        30        40        50
                                  |         |         |         |         |
Species
L. straminea      -NHNFNMNASSGFGNRNRSCLVFIGDKIYWVFYSMFRGTYFVVELYFKWW
L. hydiana        -NHNFNMNASSGFGNRNRSCLVFIGDKIYWVFYSMFRGTYFVVELYFKWW
A. ligamentina    -NHNFNVNASSGFGNRSRSCLVFIGDKIYWVFYSMFRGTYFVVGLYFKWW
L. ovata          ---NHNSNMNASSGSGDRGCLMFIGDVIYWVLYSTYRGTCFMVGLYFKWW
H. subangulata    -NHNSNTNASGSSKNLNRSYLMLIGDAVYWVFYSTYQGISFAVGLYFKWW
V. ellipsifirmis  ----NHNSNMNSGGSKNRGYFMIVGDVVYWVFSIIYEGVYISAKLYVLWW
O. olivaria       ----NHDSNMNSGGSKNRGYLMIVGDTFYWVFSIICEGIYISAKLYMLWW
P. fasciolaris    NHDSNTNAGDGGVEKKSVGLLGTLVSAIKYVFVSIFKTTFLLGKVYFLAW
L. rimosus        -NHDKNTNAGGGDVSKGGSLLGVLSSLIGYVFFGVLKATILLGKVYFLWW
G. rotundata      ---NHNMNTNSSGSSGGGSYCSGVWDIIRYVVCGIVKVAIFLGKMYGMWW
C. tampicoensis   --NHNKNANACSGPGGGGSYCSVVWDVIRYVVCGILKAAIFIGKMYGLWW
O. reflexa        ---NHNKNISHGGDENNASFLSVVRDIIGYVLRKVLGGILILGKLYVLWW
T. lividus        -----SHDKNMNAGDSGDGGRSVVWNAAVWVIRMILKVTIFCGKAYFLWW
P. popeii         -NHDKNINANCADCSCDSGFMMAVWKAIMCALGAIWKLTKFLAWLYVWWF
A. plicata        ------NHNINVNASTGGGLCGSFWDYVVYGFLGIWKCVKFLGKLYVMWF
P. dombeyanus     --NHDMNLNANASYTCSNNLCAGAWGAIVCVAKKIWGVTKFLGSCYVMWF
F. flava          --NHDENMKNMVGASNSWSVAGYAWGLLTSAAKKLLEFLKMAGTMYVMWF
P. sintoxia       ---NHDENMKNVKGANDWSVTAYAWALLTSAAKKLLELLKMAGTMYVMWF
Q. quadrula       ----NHGKDVNNSGVVDSANGFSLRGFLMGVFKKIVKVLKMLGSLYVMWF
Q. refulgens      ----NHSKDVNNSGVVDGVGGFSLRGFLMGVFKKVVKVLKMLGSLYVMWF
I. japanensis     ------NHNNVLKEMGGRPESWTWWGLLVAVVSGIGKGLYWLGSMYGMFL
```

```
                          60        70        80        90        100
                           |         |         |         |         |
L. straminea      FYLLKFGIYWPVKFVFESTFSLTTWALNTSYSLVVWFVWFLSDPVDASTS
L. hydiana        FYLLKFGIYWPVKFVFESTFSLTTWALNTSYSLVVWFVWFLSDPVDASTS
A. ligamentina    FYLLKFGIYWPVKFALESTFSLTTWALNTSYSLVVWFVWFLSDPVDASTS
L. ovata          FYFFKFGVYWPLKFTLESAFNLTSWALSTSYSLVSWFVWFLSDPVDASVS
H. subangulata    FYVLKVGIYVPLSCTLKAVFNLGQWTFNVSVSLAKWFMWFLSDPVDASLS
V. ellipsifirmis  YYFFEYCVVFPVKFALEGVYSLTSMFFKTCVSLVMWVGWFVSDPVGATVG
O. olivaria       YYFFKYGVVFPVKCALEGAYSLTSMVLKTCVSLVVWVGWFMSDPVGATVG
P. fasciolaris    YYLGYYSIYVPFSFVFLGAFDLIWWVSYICVAVGSWMSWFLTNPFDAVSF
L. rimosus        YYLGYYVVYVPVSYVFIGTFDFVWWAVSTCVAFGSWLSWFVMDPIDATMF
G. rotundata      YCFGYYVVYLPVKYTLVGTFDLTCWVASTCLAAVRWASWFVSDPVDASVY
C. tampicoensis   YCLGYYVVYLPIKYTLVGTFDLTWWISSACIAIGRWIGWFVLSPVDASVY
O. reflexa        YYFFYYAVYVPVKYAIFGTFGLTWLIIETCCSVGRWVVWFVSNPVDASIF
T. lividus        YYLAYYAIYVPIKYTFWGAFDLTWWTVVTCFSIGRWVGWFVLEPVDASVY
P. popeii         YNLFYYGLYVPIKYSVLGSIDLTWWIIMTCYAVGEWALWFLVDPVNASLF
A. plicata        YYLGYYGVYLPIKFTVCSSLDLVWWTIMSCVALGEWMGWFVTNPVDASVF
P. dombeyanus     YYLGYYGVYMPIKVAVVGSFDLVWWTVSACLSVGRWVGWFAMDPVDASVF
F. flava          YYVFYYGLYVPAKFAVTTSCDLLWWTVESCVAVVKWVGWFLTSPVDASVF
P. sintoxia       YYVFYYGLYVPAKFAVTTSYDLLWWTVESCVAVVKWVGWFLTSPVDASVF
Q. quadrula       YYVIYYGLYVPAKFAVFGGCDLIQWTLKSCLAIAEWMWWFLFSPVDASIF
Q. refulgens      YYVLYYGLYVPAKFAVFGGCDLIQWALKSCLAVAEWMWWFLFSPVDASIF
I. japanensis     YYLFYYSFYVPGKFVVVGSWGFVWWVISSSVIVVKWLVWFGNSPMEAVSY
```

```
                          110       120       130       140       150
                           |         |         |         |         |
L. straminea      AIVWLGGKAFSVIHFSVTSPVMAFVWLTKKVWSLTCLVANLPFVVFDAWM
L. hydiana        AIVWLGGKAFSVIHFSVTSPVMAFVWLTKKVWSLTCLVANLPFVVFDAWM
A. ligamentina    AVVWLGGKVFSVIRFSVTSPVMAFVWLTKKVWSLTCLVANLPFVVFDPWM
L. ovata          AIVWLKGKIFSAIYFSVTSPLTAFVWLSKKVWSLTCFMANLPFVVFDAWM
H. subangulata    AVVWLGNKFFSVIYFSVTSPLTAFVWLSKKAWSFTCFIGNLPFIVFDAWM
V. ellipsifirmis  ALVFLGDKIFSVVYFSVTSPMKAFVWLVSKACKVAWFVVNFPLFAFDAWI
O. olivaria       ALVFLVDEIFSVVYFSVTSPVKLFVWLTKKAWSVAWFMVNFPVFAFDAWI
P. fasciolaris    VFLYLVGWVWSLVYFFVTSPVSACVWLAELVWKVIYVMSNVPILSLDAWV
L. rimosus        ALFYLSSEILSLIYYCVTSPIMASVWLAKGVWKVVCVLVSVPFMTFDAFM
G. rotundata      AFLYLGSKVWSVFLFCVTSPMMASVWLAKGVWSVICVSVSFPFVVFNAWV
C. tampicoensis   AFFYLGGKVWSVVLFCVTSPVSASVWLVKSIWAVVCAGVSFPFVVFDPWM
O. reflexa        AFFYLCGKFLSGIWFVVSSPVSASVWLITGIWKMIGVVASLSFFVFDAWM
T. lividus        AFMYLGGKICSAVWFAVTSPVKASVWLVKGVWKVACAVVNFPFVVFDAWM
P. popeii         ALNYLGAKIWSIMVFAITSPVSACVLLGKGVWKVVCVVASLPFLILDAWL
A. plicata        AFFYLVDKVWSAVFFVVTKPVEASVWIIKGIWKGACAVVNFPFLVFDAWM
P. dombeyanus     AISYLGGKIWSGVCFAVTSPIAASVWVVKGVWSGICAVVSFPYVAFNALV
F. flava          VCVYLVKKVGSGIWFVVTSPVVAVKWIISGVWKGACAVANFPFLVFNAWM
P. sintoxia       ACVYLVKKVGSGIWFVVTSPVVAVKWVVSGMWKGACAVVNFPFLVFNAWM
Q. quadrula       AFGYFVGKVSSGLWFVVSSPVTAVIWLAKGVWKGVCAIVWFPLTAFEAWF
Q. refulgens      AFGYLVGKVSSGLWFVVTSPVTAFVWLVKGVWSGVCAIVWFPLTAFEAWF
I. japanensis     AVGCLVANIWSVVVFFVTSPVVAVWWLVKGFCGVVVGFVWFSLYAFEAVL
```

```
                                 160       170       180
                                  |         |         |
L. straminea       SCMSSFSDNETKQWVVMQVARSSEVFYKAMVEYYSKK
L. hydiana         NCMSSFSDNETKQWVVMQVARSSEVFYKAMVEYYSKK
A. ligamentina     DCMSSFSDNETKQWVVIQIARSSEVFYKAMVEYYSKK
L. ovata           NNMSSFSDNETKSWVVAQIARNSEVFYSAMMEYYSKK
H. subangulata     DTMSTFSGNESKRWVVTQIARNSEVFYKVMMDYYSKK
V. ellipsifirmis   DVMSSFSNNETKQWIVTHIARNTSEFYRAMVEYYSKK
O. olivaria        DVMSSFSNNETKQWIVAHIARNTSEFYRTMVEYYSKK
P. fasciolaris     SFINYYSSSETQKYIVRQIANNTKEFFDVLLKHYVKK
L. rimosus         DCMSSFSSNETKEYVVRRISKNTKEFFDVLLSYYSKK
G. rotundata       DVMSSFSYNETKEYVVRHITRNTKEFYWIMMGYYGKK
C. tampicoensis    DVMGSFSYNETKEYVVWQITRNTKEFYWIMMGYYGKK
O. reflexa         DAMSSFSDNETKEWVIWHITRNTKEFYWVVMSYYAEK
T. lividus         NVMSSFSDNDTKECVFWHITRNTKEFYWIMMGYYGKK
P. popeii          ESMSSFSDNETKSWIVWQINRNTKEFILILMGYYSKK
A. plicata         EQMSSFSSNDTKGFVVWHIYRNTKEFYWALLDRYSGK
P. dombeyanus      DSVSSFNENGVQELIAWQVYRSTKRFYWALLNRYSGK
F. flava           ESMSTFTQNETKDLVIWHVYRNTKEFIWALAERYKGD
P. sintoxia        ESMSTFTQNETKDLVVWHVYRNTKEFIWALAERYKTG
Q. quadrula        DSMSSFTDNDTKNLVVWHIYRNTKEFVWALMERYKD-
Q. refulgens       DSMSSFTDNDTKNMVVWHIYRNTKEFVWALMERYKD-
I. japanensis      NSLSSFTGDGFQEFVVDNVSWNTKKFLWILSNRYKGG
```
