# Supplementary material for: Extreme primary and secondary protein structure variability in the chimeric male-transmitted cytochrome c oxidase subunit II protein in freshwater mussels: Evidence for an elevated amino acid substitution rate in the face of domain-specific purifying selection
Source: BMC Evol Biol. 2008 May 31;8:165. doi: 10.1186/1471-2148-8-165 (PMC2430956; doi:10.1186/1471-2148-8-165)
Supplement: Additional file 2 — Pairwise amino acid and nucleotide distances (based on PC and TN models, respectively). [file 1471-2148-8-165-S2.pdf]

Additional Table 2. Pair-wise Tamura-Nei-corrected nucleotide (nu) and Poisson-corrected amino acid (aa) distances ± standard error (SE) for the taxa appearing in Table 1. Blocks 1 and 2: M*COX2*h+*COX1* in lower left and F*COX2*h+*COX1* in upper right. Blocks 3 and 4 are M*COX2*e.

Block 1:

	L.straminea	L.hydiana	A.ligamentina	L.ovata	H.subangulata	V.ellipsiformis	O.olivaria	P.fasciolaris	L.rimosus	C.tampicoensis	G.rotundata
L.str (nu±SE)	-	0.034 ± 0.008	0.080 ± 0.012	0.081 ± 0.013	0.097 ± 0.014	0.086 ± 0.013	0.079 ± 0.013	0.104 ± 0.015	0.122 ± 0.016	0.141 ± 0.018	0.108 ± 0.014
(aa±SE)	-	0 ± 0	0.005 ± 0.005	0.005 ± 0.005	0.016 ± 0.009	0.005 ± 0.005	0.016 ± 0.009	0.016 ± 0.009	0.016 ± 0.009	0.011 ± 0.007	0.005 ± 0.005
L.hyd (nu±SE)	0.013 ± 0.004	-	0.086 ± 0.013	0.099 ± 0.015	0.096 ± 0.014	0.108 ± 0.015	0.094 ± 0.015	0.113 ± 0.016	0.117 ± 0.016	0.135 ± 0.017	0.121 ± 0.015
(aa±SE)	0.003 ± 0.003	-	0.005 ± 0.005	0.005 ± 0.005	0.016 ± 0.009	0.005 ± 0.005	0.016 ± 0.009	0.016 ± 0.009	0.016 ± 0.009	0.011 ± 0.007	0.005 ± 0.005
A.lig (nu±SE)	0.049 ± 0.008	0.053 ± 0.008	-	0.095 ± 0.013	0.099 ± 0.015	0.099 ± 0.014	0.090 ± 0.013	0.116 ± 0.016	0.121 ± 0.015	0.136 ± 0.016	0.134 ± 0.016
(aa±SE)	0.010 ± 0.006	0.014 ± 0.007	-	0 ± 0	0.011 ± 0.007	0 ± 0	0.011 ± 0.008	0.021 ± 0.010	0.011 ± 0.007	0.005 ± 0.005	0 ± 0
L.ova (nu±SE)	0.114 ± 0.012	0.116 ± 0.013	0.103 ± 0.012	-	0.103 ± 0.015	0.102 ± 0.014	0.099 ± 0.014	0.118 ± 0.017	0.100 ± 0.015	0.131 ± 0.017	0.118 ± 0.015
(aa±SE)	0.028 ± 0.009	0.031 ± 0.010	0.024 ± 0.009	-	0.011 ± 0.007	0 ± 0	0.011 ± 0.008	0.021 ± 0.010	0.011 ± 0.007	0.005 ± 0.005	0 ± 0
H.sub (nu±SE)	0.110 ± 0.013	0.112 ± 0.013	0.090 ± 0.011	0.109 ± 0.012	-	0.098 ± 0.014	0.091 ± 0.013	0.120 ± 0.016	0.140 ± 0.018	0.106 ± 0.015	0.138 ± 0.016
(aa±SE)	0.042 ± 0.011	0.038 ± 0.011	0.038 ± 0.011	0.038 ± 0.011	-	0.011 ± 0.007	0.021 ± 0.011	0.032 ± 0.013	0.021 ± 0.010	0.016 ± 0.009	0.011 ± 0.007
V.ell (nu±SE)	0.149 ± 0.015	0.148 ± 0.015	0.128 ± 0.013	0.160 ± 0.015	0.133 ± 0.013	-	0.059 ± 0.010	0.126 ± 0.017	0.137 ± 0.017	0.124 ± 0.016	0.111 ± 0.014
(aa±SE)	0.093 ± 0.018	0.089 ± 0.018	0.081 ± 0.017	0.085 ± 0.018	0.096 ± 0.018	-	0.011 ± 0.008	0.021 ± 0.010	0.011 ± 0.007	0.005 ± 0.005	0 ± 0
O.oli (nu±SE)	0.139 ± 0.014	0.138 ± 0.014	0.121 ± 0.013	0.148 ± 0.015	0.124 ± 0.013	0.046 ± 0.007	-	0.105 ± 0.016	0.125 ± 0.016	0.138 ± 0.018	0.115 ± 0.014
(aa±SE)	0.074 ± 0.016	0.070 ± 0.016	0.063 ± 0.014	0.067 ± 0.015	0.078 ± 0.016	0.021 ± 0.009	-	0.032 ± 0.013	0.021 ± 0.010	0.016 ± 0.009	0.011 ± 0.008
P.fas (nu±SE)	0.163 ± 0.016	0.168 ± 0.016	0.179 ± 0.017	0.178 ± 0.017	0.163 ± 0.016	0.181 ± 0.017	0.182 ± 0.016	-	0.120 ± 0.017	0.121 ± 0.016	0.119 ± 0.016
(aa±SE)	0.127 ± 0.022	0.123 ± 0.021	0.123 ± 0.022	0.119 ± 0.020	0.111 ± 0.020	0.146 ± 0.023	0.131 ± 0.021	-	0.032 ± 0.013	0.016 ± 0.009	0.021 ± 0.010
L.rim (nu±SE)	0.186 ± 0.017	0.187 ± 0.017	0.193 ± 0.018	0.194 ± 0.018	0.175 ± 0.017	0.192 ± 0.017	0.189 ± 0.017	0.105 ± 0.012	-	0.158 ± 0.019	0.134 ± 0.016
(aa±SE)	0.135 ± 0.022	0.131 ± 0.022	0.131 ± 0.022	0.131 ± 0.022	0.108 ± 0.021	0.150 ± 0.023	0.135 ± 0.022	0.056 ± 0.013	-	0.016 ± 0.009	0.011 ± 0.007
C.tam (nu±SE)	0.187 ± 0.017	0.184 ± 0.017	0.175 ± 0.016	0.188 ± 0.017	0.151 ± 0.014	0.175 ± 0.016	0.171 ± 0.016	0.154 ± 0.014	0.184 ± 0.017	-	0.108 ± 0.015
(aa±SE)	0.119 ± 0.020	0.115 ± 0.020	0.115 ± 0.020	0.119 ± 0.020	0.111 ± 0.020	0.142 ± 0.022	0.131 ± 0.021	0.108 ± 0.019	0.123 ± 0.021	-	0.005 ± 0.005
G.rot (nu±SE)	0.188 ± 0.018	0.186 ± 0.018	0.181 ± 0.018	0.194 ± 0.018	0.162 ± 0.015	0.188 ± 0.017	0.190 ± 0.018	0.169 ± 0.016	0.169 ± 0.017	0.126 ± 0.014	-
(aa±SE)	0.093 ± 0.019	0.089 ± 0.018	0.089 ± 0.019	0.093 ± 0.018	0.089 ± 0.017	0.119 ± 0.020	0.104 ± 0.019	0.085 ± 0.017	0.100 ± 0.019	0.056 ± 0.013	-
O.ref (nu±SE)	0.173 ± 0.018	0.175 ± 0.018	0.173 ± 0.017	0.172 ± 0.017	0.148 ± 0.015	0.190 ± 0.019	0.182 ± 0.018	0.166 ± 0.016	0.170 ± 0.017	0.153 ± 0.015	0.146 ± 0.015
(aa±SE)	0.111 ± 0.020	0.115 ± 0.020	0.108 ± 0.019	0.108 ± 0.019	0.111 ± 0.019	0.142 ± 0.022	0.127 ± 0.021	0.123 ± 0.021	0.127 ± 0.021	0.119 ± 0.020	0.104 ± 0.019
T.liv (nu±SE)	0.188 ± 0.017	0.184 ± 0.017	0.201 ± 0.018	0.192 ± 0.017	0.176 ± 0.016	0.196 ± 0.017	0.195 ± 0.018	0.162 ± 0.015	0.162 ± 0.015	0.156 ± 0.015	0.178 ± 0.018
(aa±SE)	0.119 ± 0.020	0.123 ± 0.020	0.115 ± 0.020	0.111 ± 0.019	0.108 ± 0.019	0.142 ± 0.022	0.123 ± 0.020	0.096 ± 0.017	0.108 ± 0.018	0.115 ± 0.019	0.093 ± 0.016
P.pop (nu±SE)	0.219 ± 0.018	0.214 ± 0.017	0.201 ± 0.017	0.236 ± 0.018	0.198 ± 0.017	0.217 ± 0.018	0.219 ± 0.018	0.216 ± 0.018	0.224 ± 0.018	0.210 ± 0.018	0.207 ± 0.017
(aa±SE)	0.162 ± 0.024	0.158 ± 0.024	0.158 ± 0.025	0.162 ± 0.025	0.154 ± 0.024	0.191 ± 0.027	0.174 ± 0.025	0.158 ± 0.023	0.162 ± 0.023	0.162 ± 0.024	0.150 ± 0.023
P.dom (nu±SE)	0.242 ± 0.020	0.246 ± 0.020	0.232 ± 0.020	0.243 ± 0.021	0.224 ± 0.019	0.247 ± 0.020	0.243 ± 0.020	0.222 ± 0.019	0.231 ± 0.020	0.203 ± 0.018	0.208 ± 0.018
(aa±SE)	0.174 ± 0.025	0.178 ± 0.025	0.170 ± 0.024	0.170 ± 0.024	0.182 ± 0.026	0.220 ± 0.029	0.203 ± 0.027	0.170 ± 0.025	0.203 ± 0.027	0.150 ± 0.023	0.158 ± 0.023
A.pli (nu±SE)	0.219 ± 0.019	0.219 ± 0.019	0.228 ± 0.020	0.240 ± 0.020	0.199 ± 0.018	0.230 ± 0.020	0.234 ± 0.020	0.199 ± 0.018	0.199 ± 0.017	0.171 ± 0.016	0.194 ± 0.018
(aa±SE)	0.146 ± 0.021	0.142 ± 0.021	0.142 ± 0.021	0.135 ± 0.021	0.123 ± 0.020	0.182 ± 0.025	0.166 ± 0.023	0.138 ± 0.022	0.146 ± 0.023	0.142 ± 0.021	0.135 ± 0.020
P.sin (nu±SE)	0.210 ± 0.017	0.216 ± 0.018	0.212 ± 0.017	0.227 ± 0.018	0.214 ± 0.017	0.232 ± 0.019	0.238 ± 0.019	0.227 ± 0.018	0.230 ± 0.019	0.221 ± 0.019	0.232 ± 0.020
(aa±SE)	0.170 ± 0.023	0.166 ± 0.023	0.166 ± 0.024	0.166 ± 0.024	0.162 ± 0.023	0.195 ± 0.027	0.182 ± 0.025	0.186 ± 0.025	0.191 ± 0.025	0.174 ± 0.023	0.174 ± 0.024
F.fla (nu±SE)	0.238 ± 0.019	0.236 ± 0.019	0.223 ± 0.018	0.254 ± 0.020	0.211 ± 0.018	0.253 ± 0.021	0.241 ± 0.020	0.235 ± 0.020	0.231 ± 0.019	0.216 ± 0.019	0.217 ± 0.020
(aa±SE)	0.174 ± 0.024	0.170 ± 0.023	0.170 ± 0.024	0.166 ± 0.024	0.158 ± 0.023	0.195 ± 0.026	0.182 ± 0.025	0.182 ± 0.025	0.191 ± 0.026	0.178 ± 0.024	0.170 ± 0.024
Q.qua (nu±SE)	0.222 ± 0.018	0.224 ± 0.018	0.221 ± 0.018	0.243 ± 0.020	0.221 ± 0.019	0.259 ± 0.022	0.253 ± 0.022	0.235 ± 0.019	0.260 ± 0.021	0.236 ± 0.019	0.236 ± 0.020
(aa±SE)	0.174 ± 0.025	0.170 ± 0.025	0.170 ± 0.025	0.170 ± 0.025	0.174 ± 0.025	0.207 ± 0.029	0.186 ± 0.027	0.178 ± 0.026	0.199 ± 0.027	0.170 ± 0.025	0.170 ± 0.025
Q.ref (nu±SE)	0.214 ± 0.018	0.219 ± 0.019	0.219 ± 0.018	0.243 ± 0.020	0.208 ± 0.018	0.239 ± 0.020	0.233 ± 0.020	0.224 ± 0.019	0.254 ± 0.021	0.217 ± 0.018	0.254 ± 0.022
(aa±SE)	0.162 ± 0.024	0.166 ± 0.024	0.158 ± 0.024	0.166 ± 0.025	0.170 ± 0.025	0.207 ± 0.028	0.186 ± 0.026	0.191 ± 0.028	0.203 ± 0.027	0.174 ± 0.026	0.174 ± 0.026
I.jap (nu±SE)	0.313 ± 0.024	0.316 ± 0.023	0.304 ± 0.023	0.287 ± 0.022	0.299 ± 0.023	0.303 ± 0.023	0.286 ± 0.021	0.301 ± 0.022	0.322 ± 0.025	0.280 ± 0.021	0.298 ± 0.023
(aa±SE)	0.237 ± 0.030	0.241 ± 0.030	0.241 ± 0.030	0.228 ± 0.029	0.233 ± 0.030	0.259 ± 0.032	0.246 ± 0.031	0.246 ± 0.030	0.254 ± 0.031	0.220 ± 0.028	0.220 ± 0.028

Additional file 2 (continued).

Block 2:

Male	O.reflexa	T.lividus	P.popei	P.dombeyanus	A.plicata	P.sintoxia	F.flava	Q.quadrula	Q.refulgens	I.japanensis
L.str (nu±SE)	0.101 ± 0.015	0.132 ± 0.018	0.156 ± 0.020	0.131 ± 0.018	0.127 ± 0.017	0.165 ± 0.020	0.151 ± 0.019	0.147 ± 0.018	0.155 ± 0.017	0.192 ± 0.020
(aa±SE)	0.005 ± 0.005	0.011 ± 0.007	0.011 ± 0.007	0.021 ± 0.010	0.005 ± 0.005	0.021 ± 0.010	0.011 ± 0.008	0.011 ± 0.007	0.016 ± 0.009	0.016 ± 0.009
L.hyd (nu±SE)	0.123 ± 0.017	0.149 ± 0.020	0.151 ± 0.019	0.131 ± 0.017	0.129 ± 0.017	0.162 ± 0.020	0.150 ± 0.019	0.144 ± 0.017	0.155 ± 0.018	0.196 ± 0.021
(aa±SE)	0.005 ± 0.005	0.011 ± 0.007	0.011 ± 0.007	0.021 ± 0.010	0.005 ± 0.005	0.021 ± 0.010	0.011 ± 0.008	0.011 ± 0.007	0.016 ± 0.009	0.016 ± 0.009
A.lig (nu±SE)	0.105 ± 0.014	0.128 ± 0.016	0.136 ± 0.017	0.129 ± 0.017	0.133 ± 0.017	0.136 ± 0.017	0.151 ± 0.018	0.144 ± 0.016	0.147 ± 0.017	0.182 ± 0.019
(aa±SE)	0 ± 0	0.005 ± 0.005	0.005 ± 0.005	0.016 ± 0.009	0 ± 0	0.016 ± 0.009	0.005 ± 0.005	0.005 ± 0.005	0.011 ± 0.007	0.011 ± 0.007
L.ova (nu±SE)	0.032 ± 0.007	0.111 ± 0.015	0.151 ± 0.019	0.139 ± 0.019	0.132 ± 0.017	0.154 ± 0.019	0.152 ± 0.019	0.161 ± 0.019	0.149 ± 0.018	0.187 ± 0.020
(aa±SE)	0 ± 0	0.005 ± 0.005	0.005 ± 0.005	0.016 ± 0.009	0 ± 0	0.016 ± 0.009	0.005 ± 0.005	0.005 ± 0.005	0.011 ± 0.007	0.011 ± 0.007
H.sub (nu±SE)	0.112 ± 0.016	0.126 ± 0.017	0.160 ± 0.020	0.118 ± 0.015	0.123 ± 0.015	0.136 ± 0.016	0.133 ± 0.016	0.150 ± 0.018	0.153 ± 0.018	0.183 ± 0.020
(aa±SE)	0.011 ± 0.007	0.016 ± 0.009	0.016 ± 0.009	0.005 ± 0.005	0.011 ± 0.007	0.027 ± 0.012	0.016 ± 0.009	0.016 ± 0.008	0.021 ± 0.010	0.021 ± 0.010
V.ell (nu±SE)	0.107 ± 0.015	0.128 ± 0.017	0.161 ± 0.020	0.136 ± 0.016	0.140 ± 0.018	0.163 ± 0.020	0.138 ± 0.017	0.162 ± 0.019	0.141 ± 0.017	0.176 ± 0.019
(aa±SE)	0 ± 0	0.005 ± 0.005	0.005 ± 0.005	0.016 ± 0.009	0 ± 0	0.016 ± 0.009	0.005 ± 0.005	0.005 ± 0.005	0.011 ± 0.007	0.011 ± 0.007
O.oli (nu±SE)	0.113 ± 0.016	0.136 ± 0.018	0.170 ± 0.021	0.124 ± 0.016	0.133 ± 0.018	0.160 ± 0.019	0.151 ± 0.019	0.160 ± 0.019	0.147 ± 0.018	0.182 ± 0.020
(aa±SE)	0.011 ± 0.008	0.016 ± 0.009	0.016 ± 0.009	0.027 ± 0.012	0.011 ± 0.008	0.027 ± 0.012	0.016 ± 0.010	0.016 ± 0.009	0.021 ± 0.011	0.021 ± 0.011
P.fas (nu±SE)	0.128 ± 0.018	0.142 ± 0.017	0.152 ± 0.018	0.151 ± 0.020	0.124 ± 0.016	0.141 ± 0.017	0.137 ± 0.016	0.168 ± 0.020	0.156 ± 0.018	0.178 ± 0.019
(aa±SE)	0.021 ± 0.010	0.027 ± 0.011	0.027 ± 0.011	0.037 ± 0.014	0.021 ± 0.010	0.016 ± 0.009	0.027 ± 0.011	0.027 ± 0.011	0.032 ± 0.012	0.021 ± 0.010
L.rim (nu±SE)	0.115 ± 0.016	0.145 ± 0.017	0.167 ± 0.019	0.144 ± 0.017	0.148 ± 0.018	0.162 ± 0.019	0.162 ± 0.019	0.192 ± 0.022	0.171 ± 0.019	0.197 ± 0.021
(aa±SE)	0.011 ± 0.007	0.016 ± 0.009	0.016 ± 0.009	0.027 ± 0.011	0.011 ± 0.007	0.027 ± 0.011	0.016 ± 0.009	0.016 ± 0.009	0.021 ± 0.010	0.021 ± 0.010
C.tam (nu±SE)	0.129 ± 0.017	0.120 ± 0.015	0.132 ± 0.016	0.144 ± 0.017	0.140 ± 0.017	0.137 ± 0.017	0.136 ± 0.016	0.140 ± 0.018	0.129 ± 0.016	0.180 ± 0.019
(aa±SE)	0.005 ± 0.005	0.011 ± 0.007	0.011 ± 0.007	0.021 ± 0.010	0.005 ± 0.005	0.021 ± 0.010	0.011 ± 0.008	0.011 ± 0.007	0.016 ± 0.009	0.005 ± 0.005
G.rot (nu±SE)	0.128 ± 0.016	0.133 ± 0.017	0.147 ± 0.019	0.134 ± 0.017	0.128 ± 0.017	0.149 ± 0.018	0.132 ± 0.016	0.120 ± 0.016	0.140 ± 0.018	0.178 ± 0.019
(aa±SE)	0 ± 0	0.005 ± 0.005	0.005 ± 0.005	0.016 ± 0.009	0 ± 0	0.016 ± 0.009	0.005 ± 0.005	0.005 ± 0.005	0.011 ± 0.007	0.011 ± 0.007
O.ref (nu±SE)	–	0.106 ± 0.014	0.156 ± 0.020	0.136 ± 0.018	0.140 ± 0.018	0.145 ± 0.018	0.168 ± 0.021	0.146 ± 0.018	0.159 ± 0.019	0.179 ± 0.020
(aa±SE)	–	0.005 ± 0.005	0.005 ± 0.005	0.016 ± 0.009	0 ± 0	0.016 ± 0.009	0.005 ± 0.005	0.005 ± 0.005	0.011 ± 0.007	0.011 ± 0.007
T.liv (nu±SE)	0.153 ± 0.015	–	0.137 ± 0.018	0.144 ± 0.018	0.128 ± 0.017	0.146 ± 0.020	0.150 ± 0.019	0.155 ± 0.017	0.161 ± 0.018	0.173 ± 0.019
(aa±SE)	0.093 ± 0.017	–	0.011 ± 0.007	0.021 ± 0.010	0.005 ± 0.005	0.021 ± 0.010	0.011 ± 0.008	0.011 ± 0.007	0.016 ± 0.009	0.016 ± 0.008
P.pop (nu±SE)	0.200 ± 0.018	0.203 ± 0.017	–	0.177 ± 0.020	0.121 ± 0.015	0.159 ± 0.019	0.182 ± 0.020	0.157 ± 0.018	0.163 ± 0.019	0.197 ± 0.021
(aa±SE)	0.166 ± 0.023	0.119 ± 0.020	–	0.021 ± 0.010	0.005 ± 0.005	0.021 ± 0.010	0.011 ± 0.007	0.011 ± 0.007	0.016 ± 0.009	0.016 ± 0.009
P.dom (nu±SE)	0.198 ± 0.019	0.194 ± 0.017	0.220 ± 0.019	–	0.150 ± 0.019	0.157 ± 0.020	0.153 ± 0.019	0.141 ± 0.017	0.156 ± 0.018	0.193 ± 0.021
(aa±SE)	0.170 ± 0.024	0.135 ± 0.021	0.166 ± 0.025	–	0.016 ± 0.009	0.032 ± 0.013	0.021 ± 0.010	0.021 ± 0.010	0.027 ± 0.011	0.027 ± 0.011
A.pli (nu±SE)	0.182 ± 0.017	0.168 ± 0.016	0.211 ± 0.018	0.185 ± 0.017	–	0.140 ± 0.018	0.128 ± 0.017	0.135 ± 0.017	0.148 ± 0.018	0.181 ± 0.020
(aa±SE)	0.131 ± 0.020	0.096 ± 0.017	0.123 ± 0.020	0.123 ± 0.021	–	0.016 ± 0.009	0.005 ± 0.005	0.005 ± 0.005	0.011 ± 0.007	0.011 ± 0.007
P.sin (nu±SE)	0.213 ± 0.018	0.199 ± 0.018	0.241 ± 0.019	0.209 ± 0.018	0.184 ± 0.017	–	0.077 ± 0.013	0.158 ± 0.019	0.148 ± 0.018	0.166 ± 0.020
(aa±SE)	0.154 ± 0.022	0.142 ± 0.021	0.174 ± 0.024	0.150 ± 0.023	0.123 ± 0.020	–	0.011 ± 0.007	0.021 ± 0.010	0.016 ± 0.009	0.027 ± 0.011
F.fla (nu±SE)	0.217 ± 0.018	0.213 ± 0.019	0.228 ± 0.018	0.220 ± 0.019	0.198 ± 0.018	0.100 ± 0.011	–	0.154 ± 0.019	0.139 ± 0.016	0.159 ± 0.018
(aa±SE)	0.158 ± 0.023	0.131 ± 0.020	0.174 ± 0.024	0.170 ± 0.025	0.119 ± 0.021	0.049 ± 0.013	–	0.011 ± 0.007	0.005 ± 0.005	0.016 ± 0.009
Q.qua (nu±SE)	0.216 ± 0.018	0.226 ± 0.019	0.217 ± 0.017	0.220 ± 0.019	0.208 ± 0.017	0.189 ± 0.017	0.191 ± 0.017	–	0.117 ± 0.018	0.173 ± 0.020
(aa±SE)	0.170 ± 0.024	0.138 ± 0.021	0.174 ± 0.024	0.138 ± 0.023	0.138 ± 0.022	0.096 ± 0.019	0.108 ± 0.020	–	0.005 ± 0.005	0.016 ± 0.009
Q.ref (nu±SE)	0.199 ± 0.017	0.204 ± 0.017	0.234 ± 0.018	0.207 ± 0.018	0.206 ± 0.018	0.179 ± 0.017	0.193 ± 0.017	0.097 ± 0.011	–	0.190 ± 0.021
(aa±SE)	0.158 ± 0.023	0.135 ± 0.021	0.178 ± 0.025	0.142 ± 0.023	0.150 ± 0.023	0.100 ± 0.018	0.111 ± 0.019	0.017 ± 0.007	–	0.021 ± 0.010
I.jap (nu±SE)	0.279 ± 0.021	0.294 ± 0.023	0.337 ± 0.025	0.308 ± 0.025	0.304 ± 0.025	0.302 ± 0.023	0.327 ± 0.025	0.286 ± 0.022	0.268 ± 0.020	–
(aa±SE)	0.228 ± 0.028	0.207 ± 0.027	0.25 ± 0.03	0.207 ± 0.026	0.216 ± 0.028	0.22 ± 0.029	0.237 ± 0.030	0.191 ± 0.027	0.182 ± 0.026	–

Additional file 2 (continued).

Block 3

Ext-nu D±SE	L.straminea	L.hydiana	A.ligamentina	L.ovata	H.subangulata	V.ellipsiformis	O.olivaria	P.fasciolaris	L.rimosus	C.tampicoensis	G.rotundata
L.str (nu±SE)	-										
(aa±SE)	-										
L.hyd (nu±SE)	0.014 ± 0.006	-									
(aa±SE)	0.007 ± 0.007	-									
A.lig (nu±SE)	0.099 ± 0.016	0.105 ± 0.017	-								
(aa±SE)	0.065 ± 0.022	0.065 ± 0.022	-								
L.ova (nu±SE)	0.238 ± 0.030	0.248 ± 0.031	0.215 ± 0.027	-							
(aa±SE)	0.227 ± 0.042	0.218 ± 0.040	0.227 ± 0.041	-							
H.sub (nu±SE)	0.275 ± 0.031	0.283 ± 0.032	0.275 ± 0.031	0.261 ± 0.032	-						
(aa±SE)	0.398 ± 0.058	0.398 ± 0.058	0.368 ± 0.053	0.338 ± 0.052	-						
V.ell (nu±SE)	0.389 ± 0.042	0.374 ± 0.041	0.400 ± 0.041	0.420 ± 0.053	0.401 ± 0.042	-					
(aa±SE)	0.619 ± 0.074	0.619 ± 0.074	0.581 ± 0.070	0.568 ± 0.068	0.672 ± 0.076	-					
O.oli (nu±SE)	0.381 ± 0.041	0.375 ± 0.041	0.352 ± 0.036	0.373 ± 0.044	0.403 ± 0.044	0.124 ± 0.018	-				
(aa±SE)	0.581 ± 0.068	0.581 ± 0.068	0.544 ± 0.065	0.508 ± 0.062	0.672 ± 0.076	0.159 ± 0.035	-				
P.fas (nu±SE)	0.613 ± 0.060	0.626 ± 0.063	0.650 ± 0.071	0.724 ± 0.091	0.670 ± 0.070	0.659 ± 0.073	0.617 ± 0.067	-			
(aa±SE)	0.993 ± 0.105	1.012 ± 0.107	1.031 ± 0.110	0.993 ± 0.109	1.031 ± 0.113	1.012 ± 0.110	0.956 ± 0.105	-			
L.rim (nu±SE)	0.575 ± 0.063	0.571 ± 0.060	0.563 ± 0.061	0.577 ± 0.072	0.514 ± 0.049	0.536 ± 0.053	0.550 ± 0.060	0 ± 0.043	-		
(aa±SE)	0.788 ± 0.092	0.788 ± 0.092	0.804 ± 0.095	0.788 ± 0.091	0.852 ± 0.097	0.788 ± 0.092	0.788 ± 0.089	0.593 ± 0.074	-		
C.tam (nu±SE)	0.513 ± 0.059	0.509 ± 0.057	0.530 ± 0.062	0.507 ± 0.058	0.541 ± 0.065	0.476 ± 0.053	0.484 ± 0.058	0.533 ± 0.073	0.403 ± 0.046	-	
(aa±SE)	0.743 ± 0.084	0.743 ± 0.084	0.672 ± 0.078	0.700 ± 0.081	0.788 ± 0.086	0.700 ± 0.080	0.729 ± 0.081	0.773 ± 0.086	0.556 ± 0.072	-	
G.rot (nu±SE)	0.439 ± 0.045	0.431 ± 0.044	0.448 ± 0.046	0.415 ± 0.046	0.508 ± 0.056	0.446 ± 0.049	0.418 ± 0.047	0.530 ± 0.063	0.361 ± 0.038	0.194 ± 0.027	-
(aa±SE)	0.686 ± 0.081	0.686 ± 0.081	0.645 ± 0.078	0.632 ± 0.076	0.758 ± 0.084	0.632 ± 0.071	0.645 ± 0.071	0.788 ± 0.087	0.497 ± 0.068	0.253 ± 0.043	-
O.ref (nu±SE)	0.534 ± 0.059	0.524 ± 0.057	0.506 ± 0.053	0.539 ± 0.063	0.638 ± 0.109	0.499 ± 0.057	0.509 ± 0.066	0.519 ± 0.057	0.452 ± 0.049	0.336 ± 0.040	0.381 ± 0.047
(aa±SE)	0.729 ± 0.084	0.729 ± 0.084	0.700 ± 0.082	0.700 ± 0.085	0.820 ± 0.098	0.686 ± 0.077	0.743 ± 0.083	0.869 ± 0.098	0.606 ± 0.075	0.508 ± 0.067	0.532 ± 0.069
T.liv (nu±SE)	0.510 ± 0.054	0.510 ± 0.056	0.479 ± 0.050	0.476 ± 0.052	0.524 ± 0.058	0.443 ± 0.044	0.451 ± 0.047	0.502 ± 0.057	0.448 ± 0.050	0.252 ± 0.029	0.311 ± 0.037
(aa±SE)	0.659 ± 0.077	0.645 ± 0.077	0.672 ± 0.079	0.619 ± 0.075	0.758 ± 0.087	0.619 ± 0.076	0.659 ± 0.077	0.788 ± 0.089	0.593 ± 0.073	0.318 ± 0.050	0.420 ± 0.060
P.pop (nu±SE)	0.643 ± 0.069	0.661 ± 0.071	0.619 ± 0.066	0.573 ± 0.058	0.591 ± 0.060	0.632 ± 0.066	0.618 ± 0.069	0.610 ± 0.069	0.482 ± 0.045	0.460 ± 0.047	0.479 ± 0.051
(aa±SE)	0.758 ± 0.089	0.758 ± 0.089	0.758 ± 0.088	0.729 ± 0.087	0.869 ± 0.099	0.788 ± 0.088	0.820 ± 0.092	0.758 ± 0.083	0.659 ± 0.080	0.581 ± 0.074	0.619 ± 0.078
P.dom (nu±SE)	0.744 ± 0.081	0.729 ± 0.081	0.736 ± 0.093	0.710 ± 0.085	0.796 ± 0.146	0.632 ± 0.067	0.728 ± 0.090	0.747 ± 0.164	0.477 ± 0.049	0.455 ± 0.048	0.507 ± 0.053
(aa±SE)	0.852 ± 0.097	0.852 ± 0.097	0.869 ± 0.098	0.869 ± 0.095	1.012 ± 0.111	0.836 ± 0.098	0.885 ± 0.101	0.937 ± 0.103	0.729 ± 0.088	0.593 ± 0.076	0.606 ± 0.075
A.pli (nu±SE)	0.649 ± 0.072	0.639 ± 0.069	0.615 ± 0.060	0.597 ± 0.068	0.558 ± 0.055	0.510 ± 0.050	0.523 ± 0.052	0.618 ± 0.078	0.457 ± 0.053	0.377 ± 0.039	0.461 ± 0.052
(aa±SE)	0.804 ± 0.095	0.804 ± 0.095	0.788 ± 0.094	0.758 ± 0.088	0.852 ± 0.096	0.729 ± 0.086	0.773 ± 0.089	0.758 ± 0.088	0.659 ± 0.086	0.497 ± 0.065	0.593 ± 0.074
P.sin (nu±SE)	0.623 ± 0.059	0.615 ± 0.057	0.596 ± 0.057	0.621 ± 0.062	0.592 ± 0.056	0.574 ± 0.058	0.606 ± 0.064	0.633 ± 0.065	0.577 ± 0.062	0.531 ± 0.060	0.474 ± 0.049
(aa±SE)	0.836 ± 0.098	0.836 ± 0.098	0.836 ± 0.097	0.836 ± 0.099	0.869 ± 0.103	0.743 ± 0.089	0.820 ± 0.095	0.902 ± 0.103	0.836 ± 0.094	0.714 ± 0.084	0.729 ± 0.084
F.fla (nu±SE)	0.666 ± 0.064	0.657 ± 0.062	0.597 ± 0.056	0.700 ± 0.080	0.676 ± 0.068	0.599 ± 0.058	0.640 ± 0.069	0.655 ± 0.070	0.602 ± 0.065	0.638 ± 0.088	0.570 ± 0.062
(aa±SE)	0.836 ± 0.098	0.836 ± 0.098	0.836 ± 0.098	0.836 ± 0.099	0.902 ± 0.106	0.820 ± 0.095	0.885 ± 0.099	0.885 ± 0.101	0.869 ± 0.098	0.773 ± 0.088	0.758 ± 0.086
Q.qua (nu±SE)	0.622 ± 0.060	0.608 ± 0.058	0.603 ± 0.059	0.594 ± 0.059	0.560 ± 0.054	0.568 ± 0.056	0.564 ± 0.057	0.553 ± 0.054	0.551 ± 0.056	0.480 ± 0.048	0.478 ± 0.049
(aa±SE)	0.920 ± 0.109	0.920 ± 0.109	0.869 ± 0.101	0.869 ± 0.105	0.920 ± 0.108	0.869 ± 0.097	0.885 ± 0.099	0.902 ± 0.102	0.836 ± 0.100	0.700 ± 0.082	0.686 ± 0.084
Q.ref (nu±SE)	0.623 ± 0.059	0.609 ± 0.057	0.578 ± 0.053	0.562 ± 0.053	0.587 ± 0.060	0.617 ± 0.065	0.569 ± 0.057	0.597 ± 0.058	0.574 ± 0.057	0.499 ± 0.052	0.489 ± 0.047
(aa±SE)	0.804 ± 0.098	0.804 ± 0.098	0.758 ± 0.092	0.773 ± 0.094	0.836 ± 0.098	0.804 ± 0.092	0.804 ± 0.092	0.869 ± 0.095	0.804 ± 0.095	0.659 ± 0.079	0.645 ± 0.078
I.jap (nu±SE)	0.916 ± 0.140	0.887 ± 0.114	0.956 ± 0.146	0.890 ± 0.107	0.989 ± 0.237	0.854 ± 0.108	0.854 ± 0.108	0.783 ± 0.089	0.698 ± 0.071	0.738 ± 0.081	0.715 ± 0.073
(aa±SE)	1.202 ± 0.129	1.179 ± 0.125	1.249 ± 0.136	1.202 ± 0.133	1.274 ± 0.145	1.071 ± 0.122	1.092 ± 0.124	1.012 ± 0.109	0.937 ± 0.102	0.920 ± 0.104	0.937 ± 0.107

Additional file 2 (continued).

Block 4.

Ext-nu D±SE	O.reflexa	T.lividus	P.popei	P.dombeyanus	A.plicata	P.sintoxia	F.flava	Q.quadrula	Q.refulgens	I.japanensis
O.ref (nu±SE)	-									
(aa±SE)	-									
T.liv (nu±SE)	0.309 ± 0.036	-								
(aa±SE)	0.463 ± 0.064	-								
P.pop (nu±SE)	0.458 ± 0.047	0.396 ± 0.043	-							
(aa±SE)	0.593 ± 0.072	0.568 ± 0.075	-							
P.dom (nu±SE)	0.514 ± 0.060	0.472 ± 0.053	0.549 ± 0.062	-						
(aa±SE)	0.729 ± 0.089	0.568 ± 0.073	0.672 ± 0.081	-						
A.pli (nu±SE)	0.439 ± 0.048	0.394 ± 0.043	0.452 ± 0.045	0.361 ± 0.040	-					
(aa±SE)	0.593 ± 0.072	0.474 ± 0.064	0.581 ± 0.073	0.508 ± 0.070	-					
P.sin (nu±SE)	0.475 ± 0.048	0.483 ± 0.052	0.540 ± 0.054	0.492 ± 0.055	0.356 ± 0.038	-				
(aa±SE)	0.659 ± 0.081	0.606 ± 0.077	0.645 ± 0.080	0.556 ± 0.070	0.398 ± 0.058	-				
F.fla (nu±SE)	0.492 ± 0.049	0.551 ± 0.064	0.595 ± 0.067	0.465 ± 0.047	0.367 ± 0.038	0.108 ± 0.017	-			
(aa±SE)	0.632 ± 0.079	0.632 ± 0.076	0.645 ± 0.079	0.581 ± 0.070	0.398 ± 0.057	0.058 ± 0.021	-			
Q.qua (nu±SE)	0.434 ± 0.042	0.453 ± 0.047	0.470 ± 0.046	0.487 ± 0.053	0.363 ± 0.035	0.339 ± 0.037	0.355 ± 0.038	-		
(aa±SE)	0.659 ± 0.079	0.632 ± 0.075	0.672 ± 0.087	0.632 ± 0.078	0.497 ± 0.069	0.398 ± 0.063	0.409 ± 0.063	-		
Q.ref (nu±SE)	0.474 ± 0.045	0.495 ± 0.053	0.486 ± 0.049	0.492 ± 0.058	0.418 ± 0.045	0.325 ± 0.035	0.361 ± 0.038	0.119 ± 0.019	-	
(aa±SE)	0.645 ± 0.079	0.619 ± 0.075	0.632 ± 0.081	0.581 ± 0.072	0.486 ± 0.066	0.398 ± 0.060	0.420 ± 0.061	0.072 ± 0.024	-	
I.jap (nu±SE)	0.693 ± 0.068	0.873 ± 0.113	0.851 ± 0.142	0.638 ± 0.064	0.658 ± 0.068	0.611 ± 0.065	0.635 ± 0.066	0.572 ± 0.057	0.539 ± 0.054	-
(aa±SE)	1.071 ± 0.115	0.956 ± 0.103	0.937 ± 0.101	0.804 ± 0.090	0.869 ± 0.094	0.788 ± 0.088	0.836 ± 0.093	0.820 ± 0.099	0.773 ± 0.093	-
