# Supplementary material for: Extreme primary and secondary protein structure variability in the chimeric male-transmitted cytochrome c oxidase subunit II protein in freshwater mussels: Evidence for an elevated amino acid substitution rate in the face of domain-specific purifying selection
Source: BMC Evol Biol. 2008 May 31;8:165. doi: 10.1186/1471-2148-8-165 (PMC2430956; doi:10.1186/1471-2148-8-165)
Supplement: Additional file 3 — Codeml log-likelihood values and parameter estimates for four models applied to the M and F COX2+COX1 regions. [file 1471-2148-8-165-S3.pdf]

Additional Table 3. *Codeml* log-likelihood values and parameter estimates for four models applied to the M and F *COX2-COX1* regions.

Male sequences (*COX2h+COX2e+COX1*):				
Model	No. of parameters	[illegible]	*lnL*	Parameter estimates
M1a (nearly neutral)	2	6.48	-12935.84	$\hat{p}_0 = 0.853$; $\hat{p}_1 = 0.147$; $\omega_0 = 0.046$; $\omega_1 = 1$
M2a (selection)	4	6.48	-12935.84	$\hat{p}_0 = 0.853$; $\hat{p}_1 = 0.054$; $\hat{p}_2 = 0.093$
				$\omega_0 = 0.045$; $\omega_1 = 1$; $\omega_2 = 1$
M7(beta)	2	6.02	-12609.68	$\hat{p} = 0.294$; $\hat{q} = 2.823$
M8 (beta & w>1)	4	11.76	-12604.03	$\hat{p}_0 = 0.981$; $\hat{p} = 0.329$; $\hat{q} = 3.987$
				$\hat{p}_1 = 0.019$; $\omega = 1.000$
Female sequences (*COX2h+COX1*):				
Model	No. of parameters	[illegible]	*lnL*	Parameter estimates
M1a (nearly neutral)	2	11.49	-5727.65	$\hat{p}_0 = 0.979$; $\hat{p}_1 = 0.021$; $\omega_0 = 0.007$; $\omega_1 = 1$
M2a (selection)	4	11.49	-5727.65	$\hat{p}_0 = 0.979$; $\hat{p}_1 = 0.021$; $\hat{p}_2 = 0.000$
				$\omega_0 = 0.007$; $\omega_1 = 1$; $\omega_2 = 35.887$
M7(beta)	2	11.81	-5700.71	$\hat{p} = 0.081$; $\hat{q} = 3.033$
M8 (beta & w>1)	4	11.76	-5690.24	$\hat{p}_0 = 0.994$; $\hat{p} = 0.110$; $\hat{q} = 8.522$
				$\hat{p}_1 = 0.007$; $\omega = 1.000$
