# Supplementary material for: Extreme primary and secondary protein structure variability in the chimeric male-transmitted cytochrome c oxidase subunit II protein in freshwater mussels: Evidence for an elevated amino acid substitution rate in the face of domain-specific purifying selection
Source: BMC Evol Biol. 2008 May 31;8:165. doi: 10.1186/1471-2148-8-165 (PMC2430956; doi:10.1186/1471-2148-8-165)
Supplement: Additional file 4 — Summary of TreeSAAP output showing the 31 amino acid properties of MCOX2h+MCOX1, MCOX2e and FCOX2+FCOX1. [file 1471-2148-8-165-S4.pdf]

Additional Table 4. Summary of TreeSAAP output showing the 31 amino acid properties of M*COX2*h+M*COX1*, M*COX2*e and F*COX2*h+M*COX1*. Absence of selection implies neutrality.

Amino Acid Property	M sequences		M*COX2* extension		F sequences	
	1 - 3	6 - 8	1 - 3	6 - 8	1 - 3	6 - 8
Alpha-helical tendencies						neg/destab
Average number of surrounding residues		neg/destab		neg/destab		neg/destab
Beta-structure tendencies	neg/stab	neg/destab		neg/destab		
Bulkiness		neg/destab			neg/stab	neg/destab
Buriedness	pos/stab	neg/destab		neg/destab		neg/destab
Chromatographic index		neg/destab	pos/stab	neg/destab		neg/destab
Coil tendencies		neg/destab		neg/destab		neg/destab
Composition		neg/destab				
Compressibility	neg/stab			neg/destab	neg/stab	
Equilibrium constant (ionization of COOH)	neg/stab	neg/destab	neg/stab	neg/destab	neg/stab	
Helical contact area		neg/destab	pos/stab	neg/destab		pos/destab
Hydropathy		neg/destab		neg/destab	neg/stab	neg/destab
Isoelectric point	neg/stab		neg/stab			
Long-range non-bonded energy		neg/destab		neg/destab		neg/destab
Mean r.m.s. fluctuation displacement	neg/stab	neg/destab		neg/destab	pos/stab	neg/destab
Molecular volume	pos/stab	neg/destab	pos/stab			
Molecular weight		neg/destab		neg/destab		
Normalized consensus hydrophobicity		neg/destab		neg/destab	neg/stab	
Partial specific volume		neg/destab	pos/stab	neg/destab		pos/destab
Polar requirement		neg/destab		neg/destab	pos/stab	neg/destab
Polarity		neg/destab	pos/stab	neg/destab	pos/stab	neg/destab
Power to be at the C-terminal	neg/stab	neg/destab		neg/destab		neg/destab
Power to be at the middle of alpha-helix	pos/stab	neg/destab	pos/stab	neg/destab		
Power to be at the N-terminal	neg/stab	neg/destab		neg/destab		neg/destab
Refractive index	neg/stab	neg/destab	pos/stab	neg/destab	neg/stab	
Short and medium range non-bonded energy		neg/destab		neg/destab	neg/stab	
Solvent accessible reduction ratio		neg/destab		neg/destab		neg/destab
Surrounding hydrophobicity	neg/stab				neg/destab	
Thermodynamic transfer hydrohphobicity		neg/destab	pos/stab	neg/destab		neg/destab
Total non-bonded energy	neg/stab	neg/destab	pos/stab	neg/destab	pos/stab	
Turn tendencies		neg/destab		neg/destab		neg/destab
